# Supplementary material for: Cytoplasmic nanojunctions between lysosomes and sarcoplasmic reticulum are required for specific calcium signaling
Source: F1000Res. 2014 Apr 22;3:93. [Version 1] doi: 10.12688/f1000research.3720.1 (PMC4126599; doi:10.12688/f1000research.3720.1)
Supplement: Peer review history

# Peer review: Referee response for version 1

**DOI:** 10.5256/f1000research.3986.r5274

**Published:** 2014-06-27

**Authors:** Fan Zhang

**Affiliations:** 1 Department of Pharmacology and Toxicology, Medical College of Virginia Campus, Virginia Commonwealth University, Richmond, VA, USA

NAADP mediated two-phase Ca ^2+^ release responses were observed in smooth muscle cells, including pulmonary artery smooth muscle cells (PASMCs). Lysosomes and sarcoplasmic reticulum (L-SR) junctions had been proposed as the structural bases in facilitating local lysosomal Ca ^2+^ bursts (first phase) followed by whole cell Ca ^2+^ release responses from SR (second phase) through a calcium-induced calcium release (CICR) mechanism. In their studies reported here, Nicola Fameli *et al.* attempted to define the L-SR junctions in PASMCs with a nano-level resolution using electron microscopy (EM), and proceeded to build a quantitative L-SR junctional model. Unfortunately, all these efforts will add little to our further understanding of these L-SR junctional structures, if not at all, due to the lack of solid experimental EM results in reconstructing L-SR junctions, some dogmatic presumptions in L-SR model build up, the inadequacy of validation of the model and a failure to include information on the current disputes over the identity of NAADP-associated Ca ^2+^ release channels in lysosomes. Some major concerns are as follows:

 

1. Data are not convincing and lack quality. In defining L-SR junctions, the assurance of lysosome and SR identities is fundamental. Immune-gold staining has been widely applied in EM studies to ascertain different cell organelles as well as to conduct 3D reconstruction of cell structure ^1^ ^2^. However, such a standard practice was excluded in this study as a ‘precaution’ to prevent compromising lysosome membrane definition. In line with applying empirical judgments for identifying organelles to the EM data analysis, the authors extrapolated the opposing and proximal organelles adjacent to the nucleus as lysosomes and SR, and deemed them to be worth studying (Figure 2). In addition, the authors stated that there were few lysosomes found in the subplasmalemmal region, which implied that L-SR junctions were profoundly located around the nucleus. However, the fura-2 fluorescence images in Figure 1 as well as in the two citations used as basis of this study (10,11) ^3^ ^4^ exclusively demonstrated that the first phase lysosomal Ca ^2+^ release initiated from a region around the cell membrane, suggesting that L-SR junctions should have cytoplasmic locations near the plasma membrane. It is clear that more reliable methods such as immune-gold staining are needed to resolve the contradictions over their own results.
2. The modeling is based on many assumed parameters and lacks reliable validation. To establish the L-SR nanojunctional model, the present study has introduced many assumptions to some critical parameters such as the distance between the lysosome and SR, the size of lysosome, and total number of lysosomes; many of which lack proper judgments. In the abstract section, the authors rationalized the application of EM in investigating L-SR junctions by stating “L *-SR junction membrane separation has been estimated to be < 400 nm and thus beyond the resolution of light microscopy*”. In defining the L-SR nanojunctions, they declared that lysosomes and SR “ *must be separated by a distance of approximately 20 nm and be of the order of a few hundred nm in lateral dimensions*” by citing PM-SR junctions studies (10, 11) ^3^ ^4^. However, these two citations originated from the light microscope-based studies of L-SR junctions by the same group. Given the resolution limitation, these light microscopy results have no way to give an estimation as accurate as 20 nm. It is therefore confusing to find that these references fail to back up such claims, in my opinion. In reference 39 ^5^, it has been proposed that the extracellular Ca ^2+^ entered the cell through Na+/ Ca ^2+^ exchanges, traversed PM-SR junctions to refill SR via Ca ^2+^-ATPase and sustained cell Ca ^2+^ oscillations. For the current L-SR model, this PM-SR modeling may be used as a comparison or reference system leading to the conclusion “ *that L-SR had a similar 20 nm gap measured as that in PM-SR junctions*.” Given that there are no similarities of PM-SR to L-SR junctions, in terms of potency of Ca ^2+^ movement due to Ca ^2+^ gradient across PM and lysosomal membrane, biophysical property of Na ^+^/ Ca ^2+^ exchangers and TPC2 channels, and the actions of Ca ^2+^-ATPase and RyR3 Ca ^2+^ receptors, such a conclusion is not reliable from the modeling. In addition, a major concern is that this model is too simplistic as it excludes many other Ca ^2+^ regulatory mechanisms in both lysosomes and SR, which may influence the accuracy of junction width or prediction in modeling.
3. Some assumptions contradict well-established concepts due to a lack of consideration of lysosome biology. In explaining the shoulder Ca ^2+^ signal (Figure 1), the authors introduced up to four assumptions and calculated that ~60-100 lysosomes were needed to generate this shoulder peak. They verified these lysosome numbers with their TEM images that “ *can expect a total of about 110 lysosomes/cell*”. Considering these two calculations however, it is obviously problematic because almost all lysosomes (100 out of 110 each cell) will need to functionally contribute to the L-SR junctional structure formation. In addition, TEM measurements showed the lysosome dimensions (Table 1) to be major axis of 398 ± 91 nm and minor axis of 325 ± 65 nm. Given the L-SR junction model features “ *junctional membrane separation and extension are about 15 nm and 300 nm*”, it could be postulated that all the lysosomes that participated in L-SR structure formation also have to align well with the SR in order to produce a 300 nm junctional extension. Therefore, this L-SR model actually describes a scenario whereby in PASMCs the majority of lysosomes act as the components of L-SR junctions and uniformly have impeccable arrangements to oppose the SR at 20 nm apart. However, this idealistic model largely contradicts the well-established concepts that lysosomes are active organelles that dynamically exert their different functions by their movements and other activities.
4. TPCs as lysosomal Ca ^2+^ release channels are questionable. In quantifying the L-SR model, the authors arbitrarily assumed TPC2 as the only lysosomal NADDP-activated Ca ^2+^ channel by totally disregarding recent findings that TPC2 is not a lysosomal NAADP-associated Ca ^2+^ channel, but a Na ^+^ channel ^6^, ^7^ . They also ignored the contribution of other lysosomal channels or transporters that may contribute to such L-SR junction activity. This prejudice in channel selection inevitably invites doubts on the reliability of this proposed L-SR model. In addition, the authors collectively adopted electrophysiological data from other research groups without taking into consideration any incompatibilities with the model. For example, in obtaining the total number of TPC2 channels on a lysosome, the authors *“…dividing the macroscopic whole lysosome conductance, calculated from the current values reported in a recent study on TPC2-mediated Ca ^2+^ current in isolated lysosomes (48) ^8^, by the single channel conductances(45) ^9^, thus estimating that a typical lysosomal membrane may contain ~20 TPC2*”. Unfortunately, the lysosomes used in the whole lysosome conductance recording were not normal lysosomes, but lysosomes manipulated by overexpression of TPC2 and were undergoing size enlargement. With these changes, the modified lysosomes would have a much higher TPC2 conductance compared with normal lysosomes with a natural biogenesis. It is important to use a TPC2 conductance and other parameters of channels from lysosomes of normal cells without an artificial transgene for such modeling. Otherwise, the modeling prediction will be inaccurate and the relevance of such a model will be vague.

           

In conclusion; aside from the issue that the current L-SR nanojunction model fails to contribute any new insights to our understanding of the L-SR architecture, my concerns over the reliability of data and modelling parameters are rather serious – this work is therefore ‘Not Approved’ in its current version.

I have read this submission. I believe that I have an appropriate level of expertise to state that I do not consider it to be of an acceptable scientific standard, for reasons outlined above.

## References

[1] GoldbergMWFiserovaJ: Immunogold labelling for scanning electron microscopy. *Methods Mol Biol*.2010;657: 93297-31310.1007/978-1-60761-783-9_2420602226

[2] MoroneNFujiwaraTMuraseKKasaiRSIkeHYuasaSUsukuraJKusumiA: Three-dimensional reconstruction of the membrane skeleton at the plasma membrane interface by electron tomography. *J Cell Biol*.2006;174(6) : 93851-86210.1083/jcb.20060600716954349PMC2064339

[3] KinnearNPBoittinFXThomasJMGalioneAEvansAM: Lysosome-sarcoplasmic reticulum junctions. A trigger zone for calcium signalling by nicotinic acid adenine dinucleotide phosphate and endothelin-1.*J Biol Chem*.2004;279(52) : 9354319-5432610.1074/jbc.M40613220015331591

[4] KinnearNPWyattCNClarkJHCalcraftPJFleischerSJeyakumarLHNixonGFEvansAM: Lysosomes co-localize with ryanodine receptor subtype 3 to form a trigger zone for calcium signalling by NAADP in rat pulmonary arterial smooth muscle.*Cell Calcium*.2008;44(2) : 93190-20110.1016/j.ceca.2007.11.00318191199PMC3982125

[5] FameliNvan BreemenCKuoKH: A quantitative model for linking Na+/Ca2+ exchanger to SERCA during refilling of the sarcoplasmic reticulum to sustain [Ca2+] oscillations in vascular smooth muscle.*Cell Calcium*.2007;44(6) : 93565-57510.1016/j.ceca.2007.02.00117418403

[6] WangXZhangXDongXPSamieMLiXChengXGoschkaAShenDZhouYHalrowJZhuMXClaphamDERenDXuH: TPC proteins are phosphoinositide- activated sodium-selective ion channels in endosomes and lysosomes. *Cell*.2012;151(2) : 93372-38310.1016/j.cell.2012.08.03623063126PMC3475186

[7] CangCZhouYNavarroBSeoYJArandaKShiLBattaglia-HsuSNissimIClaphamDERenD: mTOR regulates lysosomal ATP-sensitive two-pore Na(+) channels to adapt to metabolic state. *Cell*.2013;152(4) : 93778-79010.1016/j.cell.2013.01.02323394946PMC3908667

[8] SchiederMRötzerKBrüggemannABielMWahl-SchottCA: Characterization of two-pore channel 2 (TPCN2)-mediated Ca2+ currents in isolated lysosomes.*J Biol Chem*.2010;285(28) : 9321219-2122210.1074/jbc.C110.14312320495006PMC2898409

[9] PittSJFunnellTMSitsapesanMVenturiERietdorfKRuasMGanesanAGosainRChurchillGCZhuMXParringtonJGalioneASitsapesanR: TPC2 is a novel NAADP-sensitive Ca2+ release channel, operating as a dual sensor of luminal pH and Ca2+.*J Biol Chem*.2010;285(45) : 9335039-3504610.1074/jbc.M110.15692720720007PMC2966118

# Peer review: Referee response for version 1

**DOI:** 10.5256/f1000research.3986.r4843

**Published:** 2014-06-18

**Authors:** Alexei Tepikin

**Affiliations:** 1 Department of Cellular and Molecular Physiology, University of Liverpool, Liverpool, UK

This is certainly an interesting study. The characterisation of lysosomal-SR junctions is novel and conceptually important. The data look convincing but I would recommend the authors to further expand the structural part of the study. It would perhaps be useful to further verify that the organelles participating in the formation of the contacts are indeed lysosomes and that the junctions are not simply the result of accidental collisions of the two organelles. Identifying/visualising tethers between the organelles (by analogy with tethers between mitochondria and the ER ^1^) would be particularly advantageous.

Modelling part of the paper is also useful. In this respect it would be valuable to clarify the Ca ^2+^ buffering capacity of lysosomes (and its possible effect on the outcomes of the model). One could also perhaps consider measuring this important parameter using isolated lysosomes.

I have read this submission. I believe that I have an appropriate level of expertise to confirm that it is of an acceptable scientific standard.

## References

[1] CsordásGRenkenCVárnaiPWalterDButtleKFBallaTMannellaCAHajnóczkyG: Structural and functional features and significance of the physical linkage between ER and mitochondria. *J Cell Biol*.2006;175(7) : 93915-92110.1083/jcb.20060401616982799PMC2064383

# Peer review: Referee response for version 1

**DOI:** 10.5256/f1000research.3986.r4530

**Published:** 2014-04-24

**Authors:** Anthony P Albert

**Affiliations:** 1 Pharmacology and Cell Physiology, Biomedical Sciences Research Centre, Division of Biomedical Sciences, St George’s, University of London, London, UK

This is an interesting and thought provoking study which simulates NAADP-dependent Ca ^2+^ transients; these are produced by release of Ca ^2+^ from lysosome stores that induce Ca ^2+^-induced Ca ^2+^ release from SR stores via stimulation of RyR3. It is increasingly apparent that answers to many issues concerning cellular Ca ^2+^ signalling will be found by recording local Ca ^2+^ events – for instance the recently identified Ca ^2+^ sparklets representing Ca ^2+^ influx through opening of plasma membrane ion channels.

Simulation studies are always difficult to review and evaluate as they must make many assumptions. However, this work is considered and may be a useful model for how other local Ca ^2+^ events might be modeled between discrete organelles. Moreover, unlike many simulation manuscripts, I find this one clear and well-written.  

Do the authors think that the use of proximity ligation assay techniques might be appropriate to measure local interactions between TPC2 and RyR3? Also, how are lysosomes refilled with Ca ^2+^ following NAADP/TPC2 activation – a role for a lysosome store-operated Ca ^2+^ influx?

I have read this submission. I believe that I have an appropriate level of expertise to confirm that it is of an acceptable scientific standard.
